# The Food4Years Ageing Network: Improving foods and diets as a strategy for supporting quality of life, independence and healthspan in older adults

**DOI:** 10.1111/nbu.12599

**Published:** 2023-01-31

**Authors:** Miriam E. Clegg, Lisa Methven, Susan A. Lanham‐New, Mark A. Green, Niharika A. Duggal, Marion M. Hetherington

**Affiliations:** ^1^ Department of Food and Nutritional Sciences University of Reading Reading UK; ^2^ Nutritional Sciences Department, School of Biosciences and Medicine, Faculty of Health and Medical Sciences University of Surrey Surrey UK; ^3^ Department of Geography and Planning University of Liverpool Liverpool UK; ^4^ MRC‐Versus Arthritis Centre for Musculoskeletal Ageing Research, Institute of Inflammation and Ageing University of Birmingham Birmingham UK; ^5^ School of Psychology University of Leeds Leeds UK

**Keywords:** ageing, diet, food products, food system, healthspan, lifespan

## Abstract

By 2050, it is predicted that one in four people in the United Kingdom will be aged 65 years and over. Increases in lifespan are not always translated into years spent in good health. Incidence rates for chronic diseases are increasing, with treatments allowing people to live longer with their disease. There is good evidence to support changes to lifestyle to maintain or improve body composition, cognitive health, musculoskeletal health, immune function and vascular health in older adults. Much research has been done in this area, which has produced significant support for foods and nutrients that contribute to improved healthspan. Yet two major barriers remain: firstly, older adult consumers are not meeting current UK recommendations for macro‐ and micronutrients that could benefit health and quality of life and secondly, the UK‐specific recommendations may not be sufficient to support the ageing population, particularly for nutrients with key physiological roles. More work is needed to improve intakes of specific foods, diets and nutrients by older adults, through a variety of mechanisms including (i) development of specific food products; (ii) improved clarity of information and (iii) appropriate marketing, and policy changes to enable incentives. The Food4Years Ageing Network aims to build a wide‐reaching and multidisciplinary community that is committed to the development, integration and communication of healthy, affordable foods and specific diets for all older adults across the UK food landscape. The Network will identify evidence‐based strategies for improving food intake and nutrition in older adults, paving the way to “living well while living longer.”

## IMPROVING HEALTHSPAN IN THE OLDER ADULT POPULATION

It is forecast that by 2050 one in four people in the United Kingdom will be aged 65 years and over (Office for National Statistics, 2021). This demographic shift has prompted a new concept of adding “life to years,” which aims to look beyond focusing entirely on extending life years to promote better wellbeing and quality of life as we age (World Health Organization, 2020). Whilst UK life expectancy has increased through the 20th and 21st centuries, there is little evidence that these gains in life expectancy are always translated to increased years of living in good health for older adults when compared to previous generations (Lafortune et al., 2007; Robine et al., 2009). Incidence rates for diet‐related chronic diseases such as osteoporosis and type 2 diabetes are increasing (Caspersen et al., 2012; Cooper et al., 2011). Treatments that extend life rather than prevent or remedy conditions mean that people live with their disease for longer (Public Health England, 2018a). This increases health and social care costs placing additional burdens on health systems and inadvertently negatively impacting the quality of life (Dorrington et al., 2020). Consequently, increases in lifespan appear to be surpassing increases in years spent in good health, otherwise known as healthspan (Calder et al., 2018; House of Lords, 2021).

The World Health Organization (WHO) has been active in the development of the concept of adding life to years, whilst acknowledging that there is no typical older person. The diversity of health outcomes in older people is not random with people's physical and social environments and the impact of these environments on their opportunities and health behaviour playing a large role. This makes it particularly difficult to target policies for this age group. The WHO Active Ageing Policy Framework brought together leading experts to identify strategies for promoting healthy ageing, which has had a lasting impact on policy development on ageing worldwide (World Health Organization, 2002). The WHO in this Policy Framework defines Active Ageing as the “process of optimizing opportunities for health, participation and security in order to enhance quality of life as people age.” Thus, it embraces a strong life course approach, focusing on early preparation for lifelong health but with the caveat that it is never too late to get started. This has been further acknowledged in the United Nations “Decade of Healthy Aging” plan (2021–2030; World Health Organization, 2022) that brings together governments, civil society, international agencies, professionals, academia, the media and the private sector to improve the lives of older people, their families and the communities around the world to encourage lifelong health and wellbeing. Crucially, appropriate dietary choices and maintaining healthy dietary patterns are key components of this report.

## THE ROLE OF DIET IN IMPROVING HEALTHSPAN

A nutritious diet is recognised as essential for healthy ageing, wellbeing and reducing the risk of chronic diseases and the rate of functional decline (Dorrington et al., 2020). Collaborative, interdisciplinary nutrition research aims to understand what is needed to eat better and to extend life years lived well. This is achieved through complex and nuanced relationships with macro‐ and micronutrients of which we detail our current understanding below.

Recent studies have highlighted many of the benefits that diet can have for healthy ageing. However, much of the evidence is new and the benefits are not well accepted. For example, in the area of protein intake, protein has been highlighted for its ability to reduce the risk of frailty, however, sufficient energy intake is also required for the protein to stimulate muscle protein synthesis (Carbone et al., 2019; Mendonça et al., 2019). In a systematic review and meta‐analysis, protein intake also decreased hip fractures and showed a positive trend between higher protein intakes and higher femoral neck and total hip bone mineral density (BMD; Groenendijk et al., 2019). A preliminary, cross‐sectional study examining the relationship between energy intake and cognitive decline has shown that high protein intake is associated with a reduced risk of mild cognitive impairment (MCI) or dementia (Fernando et al., 2018). A double‐blind, randomised, placebo‐controlled trial in participants aged 55 years and over demonstrated that ingestion of seven essential amino acids led to improved attention and cognitive flexibility and psychosocial functioning, which could then prevent cognitive decline (Suzuki et al., 2020). Sarcopenia, a progressive and natural loss of muscle mass and strength as people age (Cruz‐Jentoft et al., 2019), results in an increased risk of falls and fractures, as well as reduced independence and exercise ability, contributing to a considerable reduction in quality of life (Hunter et al., 2019). Increasing protein intake in older adults may therefore be an effective intervention to offset muscle mass decline and improve quality of life (Ispoglou et al., 2021), particularly when combined with exercise (Walker et al., 2011). However, whilst there have been many intervention studies that have achieved an increase in protein intake (e.g. Beelen et al., 2017; Hone et al., 2022), there is very little evidence on how to increase protein intake effectively at a population level in older adults (Lonnie et al., 2018). Even when high‐protein products are developed, they are often not co‐created with older adults or they have been targeted at younger adults such as athletes and may not have the attributes required by older adults such as being low in saturated fat, micronutrient dense or easy to open and consume. Challenges remain to encourage the intake of protein‐rich foods in a population with loss of appetite (anorexia) and rapid satiation. Furthermore, protein is often considered the most satiating macronutrient (Paddon‐Jones et al., 2008) which could reduce intake further, though recent evidence from a systematic review and meta‐analysis suggests that whilst appetite ratings may be suppressed with acute protein supplementation, there is either a positive effect or no effect on total energy intake in both acute and longitudinal studies in older adults (Ben‐Harchache et al., 2021).

It is well known that cognitive function declines with age and up to 50% of those with MCI are predicted to develop dementia within 5 years (Gauthier et al., 2006). Evidence in this area implicates deficiencies of certain nutrients in cognitive decline as well as demonstrates that optimal nutritional status may preserve cognition in older adults (Moore et al., 2018). Higher intakes of fish or fruits and vegetables, as well as certain dietary patterns, particularly the Mediterranean diet (olive oil, fruit, vegetables, wholegrains and fish), have been linked with improved cognitive health (Barberger‐Gateau et al., 2007; Kang et al., 2005; Staubo et al., 2017), as well as lowering the risk factors for cardiovascular disease and improving immune health (Camargo et al., 2012; Mena et al., 2009). However, only 27% of adults aged 65 years and over consume five fruit and vegetable portions a day (Health Survey for England, 2019), and the average daily intake (g/day) of oily fish rich in polyunsaturated fatty acids (PUFA) are low in the 65 years and over age group (for men and women, 17 and 12 g for those age 65–74 years, reducing to 12 and 8 g, respectively, for those aged 75 years and over, compared to the recommended 20 g/day through one 140 g portion per week; Public Health England, 2018b). Specific nutrients have also been linked to the protection of cognitive functions with ageing including omega‐3 PUFAs, polyphenols, vitamin D and vitamin B (Annweiler et al., 2013; Brickman et al., 2014; Hooshmand et al., 2014; Mazereeuw et al., 2012; Miller et al., 2015; Moore et al., 2018).

Ageing is also accompanied by changes to the immune system, resulting in an impaired ability to mount a robust immune response, termed “immunosenescence” (Conway & Duggal, 2021; Duggal, 2018) and an elevated basal level of pro‐inflammatory cytokines, termed “inflammaging” (Franceschi et al., 2018). Both immunosenescence and inflammaging contribute towards the age‐associated increased risk of infections, chronic diseases, autoimmunity, impaired vaccine responses and poor health in older adults; addressing these could contribute towards bridging the gap between healthspan and lifespan. Improving nutritional intake has been demonstrated to maintain or enhance immune function (Clements et al., 2017; Ostan et al., 2016). Advancing age is accompanied by a loss of beneficial commensal microbes and expansion in pathobionts, resulting in a state of microbial dysbiosis (Biagi et al., 2016) which could potentially increase the risk of frailty (Mello et al., 2016). For example, faecal samples from 728 twins aged 42–86 years indicated that frailty was negatively associated with alpha diversity of the gut microbiota (Jackson et al., 2016). Although much of the human data to support this relationship are observational, animal work has highlighted that the microbiome plays an important role in the development of the immune system. For example, evidence from aged germ‐free mice has led to the postulation of the hypothesis that age‐associated microbiome changes are a potential contributor towards age‐associated immunosenescence (Conway & Duggal, 2021). Furthermore, what is becoming increasingly clear is that a healthy diet is a key contributor towards a diverse gut microbiota, which in turn is associated with a healthier immune system and overall, a healthier ageing trajectory (Claesson et al., 2012). Thus, it is no surprise that dietary supplements that promote the expansion of beneficial microbiota may prove useful for maintaining immune health in older people. Interestingly, trials providing probiotic supplementation in older adults, in addition to an immunoenhancing effect (Maneerat et al., 2013), have reported a reduced risk of respiratory infections (Guillemard et al., 2010) and improved vaccine responses (Boge et al., 2009).

Maintaining physical activity combined with a healthy weight, and ensuring recommended intakes of calcium and vitamin D, can slow the rate of bone loss (Dawson‐Hughes et al., 1997). Protein intake again plays a role as BMD has been shown to be positively associated with dietary protein intake (Rizzoli et al., 2018). Loss of BMD increases the risk of osteoporosis, which, by the age of 80 years, is observed in more than 50% of women and 10% of men. Osteoporosis impacts on quality of life and independence in older adults. Dietary changes can help to prevent or delay the effects of osteoporosis, and therefore represent a key area for future research.

There is, therefore, evidence that changes to lifestyle (i.e. diet, nutrition and physical activity) can maintain or improve body composition, cognitive and mental health, immune function and vascular health in older adults. However, two major barriers remain. Firstly, older adult consumers are not meeting current UK dietary recommendations for both macro‐ and micronutrients, yet this would provide benefits to health and quality of life. Secondly, the UK‐specific dietary recommendations for older adults and the current body of evidence to support changing these recommendations are not sufficient to support healthy ageing, particularly for nutrients with key physiological roles (Dorrington et al., 2020; Public Health England, 2021).

## NUTRITIONAL REQUIREMENTS AND OLDER ADULTS: WHERE EVIDENCE AND GUIDELINES ALIGN AND WHERE THERE ARE GAPS

Several studies have reported that energy, macro‐ and micronutrient intakes are typically lower than recommended in older people (Mendonça, Hill, et al., 2016; Mendonça, Mathers, et al., 2016). For example, data from the UK *National Diet and Nutrition Survey* from years 7 (2014–2015) and 8 (2015–2016) show that men and women aged 65–74 years are consuming, on average, just 1940 and 1483 kcal, respectively. This further reduces to 1824 and 1344 kcal for those over 75 years (Public Health England, 2018b). The Estimated Average Requirements for energy are 2342 and 1912 kcal for males and females aged 65–74 years and 2294 and 1840 kcal for males and females aged 75 years and over (Scientific Advisory Committee on Nutrition, 2011) based on an assumed average Physical Activity Level of 1.49. In the longitudinal *SENECA* study, 247 Danish and Dutch people aged 70–75 years were first surveyed and then re‐examined 4–5 years later. Results showed a significant decline in energy intake over the 4 years of follow‐up (Schroll et al., 1997). However, it is acknowledged that under‐reporting is an issue in all nutritional surveys including in older adults (Public Health England, 2018b) and data on energy intake is not consistent with data on levels of obesity, which in the United Kingdom lie at 36% of adults aged 65–74 years and 26% of adults aged 75 years and over (Health Survey for England, 2019). Nevertheless, a recent study by Sulmont‐Rossé et al. (2022) looked at the prevalence of undernutrition in older people (65 years and over) living with overweight and obesity by completing a secondary analysis of data collected through two French surveys. It indicated that 2% of the respondents with a body mass index over 25 kg/m^2^ were undernourished, but that 18% of those overweight and 29% of those with obesity were at risk of undernutrition using the Mini‐Nutritional Assessment (MNA) Screening tool. The MNA consists of 18 items including anthropometric measurements as well questions on diet, appetite, feeding, health, anthropometrics and disabilities.

Contrary to common belief, nutritional needs only decrease marginally with age and are sometimes higher than the needs of younger individuals. For example, the current UK population protein recommendation is 0.75 g/kg bodyweight/day. However, the European Society for Clinical Nutrition and Metabolism (ESPEN) and the *PROT‐AGE* Study Group have advised that a healthy older adult's recommended daily protein intake should be increased to 1–1.2 g/kg (Bauer et al., 2013; Deutz et al., 2014), to maintain functionality, independence and fight infection. This is further elevated for frail older adults who should consume 1.2–1.5 g/kg (Bauer et al., 2013). Within this recommendation, there are still unanswered questions about how protein should be consumed. Most research suggests that there is a minimum threshold for protein intake to maximise muscle protein synthesis, with two to three meals a day, each containing around 25–30 g of high‐quality protein being optimal (Farsijani et al., 2017; Loenneke et al., 2016). Research also suggests that much higher doses of protein (e.g. 30 vs. 90 g) will have a limited impact on skeletal muscle protein synthesis in younger and older participants alike (Symons et al., 2009) although older participants require a higher relative protein intake compared to younger participants to maximally stimulate muscle protein synthesis (Moore et al., 2015). Nevertheless, research suggests that older adults are not meeting this 25–30 g/meal threshold (Lonnie et al., 2018; Mendonça et al., 2018; Morris et al., 2020). In UK adults aged 70 years and over, only 30.5% of females and 40.7% of males are reaching the 1.0 g/kg target and 15% of both sexes are reaching the 1.2 g/kg target, making them vulnerable to a decline in muscle mass, frailty and loss of immune function (Green et al., 2020). The *Newcastle 85+* study, which focussed on community‐living adults aged 85 years and over, found a median protein intake of 0.97 (0.77–1.24) g/kg of adjusted bodyweight per day (Mendonça et al., 2018). Similar findings from the same dataset have indicated both men and women aged 85 years and over do not meet micronutrients such as vitamin D's Reference Nutrient Intakes with many not even reaching the Lower Reference Nutrient Intake for nutrients such as magnesium, potassium, selenium and vitamin B12 (Mendonça, Hill, et al., 2016; Mendonça, Mathers, et al., 2016). It is clear that diet quality, with a focus on selecting micronutrient‐rich and high‐protein sources, should become a priority with ageing. Helping older adults to meet their recommended nutrient intakes needs a key policy approach to improve healthspan.

A new policy framework is needed to apply the advances in scientific research including discoveries about the role of essential nutrients and non‐essential nutrients (e.g. bioactives), for healthy ageing. Advances in the evidence must inform public health policy to promote healthy ageing. However, the most recent Scientific Advisory Committee on Nutrition (SACN) position statement on nutrition and older adults living in the community did not suggest any changes to nutrient requirements specific to this age group (Public Health England, 2021). Taking protein as an example, the SACN report concludes that for the meta‐analysis that existed there was no significant effect of protein supplements on measures of musculoskeletal health in older adults. It was also highlighted that there was considerable heterogeneity in interventions, population groups and outcome measures and many of the reviews included emphasised that the studies included were of a low quality. The evidence for dietary protein and musculoskeletal health in the SACN report suggested some evidence for an association between low dietary protein intakes and a higher prevalence of frailty, however, these were based primarily on cross‐sectional studies. This highlights the need for more high‐quality studies to support changes in policy.

Much research has been done in the area of older adult nutrition and health, which has produced significant support for the types of foods and nutrients that contribute to improved health with ageing and has been imperative for constructing a data‐driven evidence base to identify potential products that might offer greater choice to consumers (Green et al., 2020; Lonnie et al., 2018). However, the SACN report also highlights large gaps in evidence across a number of areas including, but not limited to, nutrition and health in adults aged 85 years and over, nutrition in older adults from Black, Asian and minority ethnic groups, the role of dietary patterns and/or specific nutrients on cardiovascular health, cancer, gastrointestinal tract health, eye health, hydration, skin and wound healing, appetite control and energy balance, oral health and immune health in older age groups. Significant to the development of the UK's future food landscape are the recommendations to the government in the National Food Strategy Report published in July 2021 (National Food Strategy, 2021). This contains recommendations to address the major issues facing the food system including climate change, biodiversity loss, land use, diet‐related disease, health inequalities, food security and trade. Within this report there is a strong focus on children and youth, suggesting less focus on the needs of our ageing population. Until there is a shift in our policy landscape, there is a mismatch between research on older adults' nutritional needs, with a focus on meeting their specific dietary requirements and the current food system. Furthermore, the market offer of real food solutions for older adults is still modest and the food industry itself recognises the need to address the challenge of designing and marketing high‐quality foods and meals for the older adult consumer (Wiltshire Farm Foods, 2022).

## CHALLENGES TO THE DEVELOPMENT AND MARKETING OF FOOD PRODUCTS DESIGNED FOR OLDER CONSUMERS

Numerous, interacting risk factors predict malnutrition, including social, physical, medical and psychological changes in the ageing process (Norton et al., 2021). The challenges faced by older adults in terms of meeting nutrient requirements are outlined in detail in other reviews (Clegg & Williams, 2018). In brief, poor appetite is recognised as a major determinant of undernutrition (Van Der Pols‐Vijlbrief et al., 2014), as reduced intake ultimately provides fewer opportunities to consume the necessary nutrients. There is a range of challenges associated with the ageing process that can complicate food intake in older adults. For example, taste and aroma perception decline with age (Doty & Kamath, 2014; Methven et al., 2012), hence food can be perceived as less flavour intense leading to reduced appeal and intake. In addition, older adults can experience texture perception changes (Hall & Wendin, 2008; Kremer et al., 2007; Withers et al., 2013), reduced appetite (Pilgrim et al., 2015) and up to 25% of older adults have swallowing difficulties (Thiyagalingam et al., 2021). Reduced saliva production and the texture of foods can increase processing time in the mouth, promoting feelings of satiety (Chamber, 2016; Clegg & Williams, 2018) and influencing appetite regulation (Norton et al., 2021). Eating capability declines with age, and so oral processing, especially for edentulous older adults becomes more challenging (Laguna et al., 2016), leading to food selection based more on ease of eating rather than nutritional content. Loss of strength and dexterity can make packaging difficult to open and labelling of food products in font sizes that are difficult to read results in an avoidance of certain foods (Yoxall et al., 2010). Evidence suggests that once significant weight loss has occurred, aggressive nutritional support may fail to improve outcomes, hence maintenance of nutrient‐dense food intake is of considerable importance in ageing (Dent et al., 2019).

Healthy ageing is an equity issue. People of lower socio‐economic status tend to live both live shorter lives and spend a larger proportion of their lives in poor health or disability (Parker et al., 2020). Socio‐economic status is also a key determinant of dietary behaviours, with people of lower socio‐economic status less likely to meet dietary recommended values or consume healthier foods (Maguire & Monsivais, 2015). Older adults living in care homes have a greater risk of malnutrition if those care homes are situated in areas of deprivation (Norris et al., 2011). The co‐occurrence of poor diet and poor health outcomes in disadvantaged communities means that we need to develop equitable and affordable strategies to support healthy ageing.

It appears that most older adults are unaware of specific dietary needs that might be more important with ageing. In focus groups with UK older adults, Whitelock and Ensaff (2018) found that UK older adults were strongly focused on dieting, particularly removing sugar and fat from their diets. This contrasts with findings of malnutrition in UK older adults. Estimates suggest 1.3 million people aged 65 years and over suffer from malnutrition with 93% of these individuals living in the community and 32% of people aged 65 years and over in the United Kingdom are at risk of malnutrition on admission to hospital (BAPEN, 2014). However, the double burden of malnutrition (i.e. the coexistence of undernutrition along with overweight and obesity) commonly occurs in older adults (Kobylińska et al., 2022; Sulmont‐Rossé et al., 2022), resulting in a further set of chronic conditions such as sarcopenic obesity (Roh & Choi, 2020). This indicates that further research is needed to explore how to build consumer awareness about the importance of eating specifically for healthy ageing, taking into consideration the potentially confusing messaging that may evolve from asking people to lose weight whilst increasing their nutrient intakes. Improved clarity of messaging may help encourage adequate intakes of specific foods, diets and nutrients, with a shift away from dieting for weight loss towards achieving a healthy, balanced diet for wellbeing. However, such a shift needs to be supported by policymakers and national guidelines to be effective.

Older adults have very specific food needs, not just in terms of nutritional composition and physical properties of the food but also because of a reduced ability to prepare and eat foods due to declining functionality, dexterity and eating capability (Ibrahim & Davies, 2012). The food industry needs to play a role in the development of novel food products specifically for older adults, as such engagement with the food industry is key to designing, producing and marketing new products for older adult consumers. Currently, the offering of food products designed specifically for older adults is limited, particularly in mainstream supermarkets. A search of the largest UK food retailer's webpage reveals no food‐related products when searches are completed for “ageing,” “older adults” or “elderly.” In contrast, a search of other age groups known to also have specific nutritional needs, such as infants and children, reveals more than 100 food‐related products. Extracted data from the *Food and You Survey Wave 5* demonstrates that the vast majority (up to 97%) of older adults do their food and grocery shopping at large supermarkets, with the second and third highest being mini supermarkets (up to 41%; Smith et al., 2022). This indicates that for foods to be accessible to older adults they need to be widely available on our supermarket shelves. The challenge for the market is the low social collective identity among older adults and the disparity in health, socio‐economic and lifestyle factors within this group, who do not necessarily see themselves as the target segment for “older adult nutrition.”

## CO‐CREATING FOOD‐BASED SOLUTIONS WITH OLDER ADULTS

The involvement of older adults, their families and carers, industry, healthcare professionals and across demographic and socio‐economic groups is required to develop foods and diets for older adults. Older adults have very specific food requirements both in terms of nutritional composition and the physical properties of the food, food packaging and preparation (Ibrahim & Davies, 2012). These needs and requirements will vary across diverse communities. However, in contrast to items such as skincare products for ageing, which are widely marketed and purchased, the market demand for ageing‐related food products appears to be limited.

As well as the biological changes that accompany ageing, it is a period of social and psychological transition (Edstrom & Devine, 2001). Accounting for social and environmental determinants of nutrition and physical activity behaviours during ageing is a prerequisite in any intervention development (Mcnaughton et al., 2012). Sociodemographic factors are strong predictors of food choice and food fussiness with ageing, demonstrating that a one‐size‐fits‐all model will not apply (Appleton et al., 2017; Grasso et al., 2019). For example, older adults with a poor appetite and lower level of protein intake are characterised by having an education below tertiary level, some or severe financial difficulties, having less knowledge about dietary protein and being fussier about food (Hung et al., 2019). This research indicates that foods and diets need to be flexible and affordable. Furthermore, in the UK retired adults' median yearly income (£25 434) is less than that of non‐retired adults (£32 141; Office for National Statistics, 2020), which highlights that food products must be affordable for older adults to have maximum reach and impact. This is imperative as healthier foods tend to be more expensive than less healthy foods (Morris et al., 2014) suggesting that interventions to promote their consumption may exacerbate social inequalities.

Considering why and how older adults make food choices are important factor when creating foods. There are conflicting results on whether health is a key factor in food‐based decisions. Some studies indicate that taste and sensory appeal predominate choices (Kamphuis et al., 2015; Locher et al., 2009; Whitelock & Ensaff, 2018); however, eating to improve quality of life has been reported to be a motivator for older Irish adults to consume a healthier diet (Delaney & Mccarthy, 2011). Similarly, a study from The Netherlands looking at major motivators of food choice in older adults from both high and low socio‐economic groups identified healthiness as the most important attribute for all participants (Kamphuis et al., 2015). Research using focus groups indicated that foods were chosen and eaten based on enjoyment rather than anything else and were regarded as an earned entitlement now that people were older (Whitelock & Ensaff, 2018). This indicates that for food products to benefit older adults, nutrients must be added to the foods that older adults already enjoy and consume most frequently. Older adults tend to keep the same foods on rotation in their diets, rarely stray from these and do not want to change their grocery habits indicating that there may be opportunities for fortification through the incorporation of nutrients into the most commonly consumed foods (Van Der Zanden et al., 2014; Whitelock & Ensaff, 2018). Supplementation by stealth, via existing product lines, avoids the medicalisation of drinking nutritional supplements with the potential perception of ageing as an illness. Interestingly, older adults with a reduced appetite have different food preferences to those with a better appetite, preferring food and food colour variation, non‐dairy, high‐fibre and solid texture foods (Van Der Meij et al., 2015; Whitelock & Ensaff, 2018) indicating that a one‐size‐fits‐all solution may not be possible.

## THE FOOD4YEARS AGEING NETWORK

The overall aim of the Food4Years Ageing Network is to build a community that is committed to the development, integration and communication of healthy, affordable foods and specific diets for all older adults across our food landscape. This will be achieved via the following objectives:

- Build, sustain and develop a community of consumers, researchers and businesses committed to the research and development of foods and food environments that support healthy ageing.
- Coordinate the actions/events of Food4Years with relevant national bodies to disseminate key outputs.
- Increase the range of businesses, charities, clinicians and academic disciplines engaged in the development of foods and food environments that focus on nutrition for lifelong health.
- Identify research opportunities for the consumption of diets and foods specifically for older adults, at an individual (consumer), local (care home and lunch clubs) and industry levels.
- Pump‐prime new collaborations between businesses, charities and academics and help them co‐develop (i) interventions to communicate the nutritional requirements of older adults and (ii) food products and marketing strategies for older adults that are acceptable to their needs.
- Encourage early career researchers into the sector and provide funds to help engage new investigators to work with charities and industry partners.
- Stimulate public enthusiasm for lifelong nutrition by highlighting the benefits through popular media in a timely fashion in response to current news and events.
- Promote and facilitate the interaction between researchers, industry and charities, resulting in major funding awards from the United Kingdom and international sources for relevant research, which can significantly advance the developments in the field.
- Inform and influence through the development of a white paper outlining the policy requirements needed to stimulate action and integrate research into changes within our food system that can have a wide‐reaching impact to promote quality of life and added life years in the ageing UK population.

Academics, healthcare professionals, industry representatives and all those interested in older adult nutrition and health are encouraged to engage with the Network and provide their thoughts and opinions to inform the future direction of nutrition for older adults in the United Kingdom (https://www.ukanet.org.uk/food4‐years‐food‐for‐added‐life‐years‐putting‐research‐into‐action/).

The outcome of the network will be to build and apply an evidence base to identify strategies for improving food intake and nutrition in older adults, paving the way to living well whilst living longer.

## CONFLICTS OF INTEREST

There are no conflicts of interest associated with this manuscript.

## FUNDING INFORMATION

This Network was funded by the UK BBSRC and MRC (grant reference BB/W018349/1).

## Data Availability

Data sharing not applicable to this article as no datasets were generated or analysed during the current study.
